# Does availability of physical activity and food outlets differ by race and income? Findings from an enumeration study in a health disparate region

**DOI:** 10.1186/1479-5868-9-105

**Published:** 2012-09-06

**Authors:** Jennie L Hill, Clarice Chau, Candice R Luebbering, Korine K Kolivras, Jamie Zoellner

**Affiliations:** 1 Department of Human Nutrition, Foods and Exercise, Integrated Life Sciences Building 23, Room 1033 (0913), 1981 Kraft Drive, Blacksburg, VA, 24061, USA; 2 Department of Human Nutrition, Foods and Exercise, Integrated Life Sciences Building 23 (0913), 1981 Kraft Drive, Blacksburg, VA, 24061, USA; 3 Department of Geography, 115 Major Williams Hall, Blacksburg, VA, 24061, USA; 4 Department of Geography, 123 Major Williams Hall, Blacksburg, VA, 24061, USA; 5 Department of Human Nutrition, Foods and Exercise, Integrated Life Sciences Building 23, Room 1034 (0913), 1981 Kraft Drive, Blacksburg, VA, 24061, USA

**Keywords:** Built environment, Health disparities, Community-based participatory research, Spatial autocorrelation

## Abstract

**Background:**

Low-income, ethnic/racial minorities and rural populations are at increased risk for obesity and related chronic health conditions when compared to white, urban and higher-socio-economic status (SES) peers. Recent systematic reviews highlight the influence of the built environment on obesity, yet very few of these studies consider rural areas or populations. Utilizing a CBPR process, this study advances community-driven causal models to address obesity by exploring the difference in resources for physical activity and food outlets by block group race and income in a small regional city that anchors a rural health disparate region. To guide this inquiry we hypothesized that lower income and racially diverse block groups would have fewer food outlets, including fewer grocery stores and fewer physical activity outlets. We further hypothesized that walkability, as defined by a computed walkability index, would be lower in the lower income block groups.

**Methods:**

Using census data and GIS, base maps of the region were created and block groups categorized by income and race. All food outlets and physical activity resources were enumerated and geocoded and a walkability index computed. Analyses included one-way MANOVA and spatial autocorrelation.

**Results:**

In total, 49 stores, 160 restaurants and 79 physical activity outlets were enumerated. There were no differences in the number of outlets by block group income or race. Further, spatial analyses suggest that the distribution of outlets is dispersed across all block groups.

**Conclusions:**

Under the larger CPBR process, this enumeration study advances the causal models set forth by the community members to address obesity by providing an overview of the food and physical activity environment in this region. This data reflects the food and physical activity resources available to residents in the region and will aid many of the community-academic partners as they pursue intervention strategies targeting obesity.

## Background

The prevalence of obesity continues to rise in the United States and is widely recognized as a major public health concern [1]. Low-income, ethnic/racial minorities and rural populations are at increased risk for obesity and related chronic conditions (e.g. diabetes, CVD) when compared to white, urban and higher-socio-economic status (SES) peers [2-4]. This continued rise in obesity rates and persistent disparities among sub-populations has led to renewed calls for efforts to eliminate disparities and achieve equity in health for all groups [5].

It is increasingly evident that efforts focused on changing individual behavior alone are not enough to reverse the current trends in obesity [6,7]. Recently, terms such as ‘obesogenic’ are being used to describe environments in which influences beyond individual behavior are thought to contribute to the rise in obesity [8-10]. Built environments can be defined as the physical or human-made features of a neighborhood, roadways, buildings, food sources and recreation spaces [11,12]. Features of the built environment that facilitate or hinder healthy lifestyles such as physical activity and food choices include land-use, walkability, and available space and resources for activity [13].

In health research, there is a long history and supporting evidence for the influence of ‘place’ on health [14]. A relevant concept for public health research is that of deprivation amplification. In brief, deprivation amplification posits that persons who are poor, tend to live in areas that are poor, and are exposed to poor environments that negatively impact health outcomes across the lifespan [14-16]. When applied to the built environment and obesity, deprivation amplification may result in fewer resources such as parks or trails that support physical activity in low-income or racially diverse areas [17-22]. Further, in low-resource areas these facilities may not be safe, may contain poorly maintained equipment or amenities or may not be aesthetically appealing for outdoor activity, thereby inhibiting physical activity such as walking or park and trail use [23-27]. In the food environment literature recent reviews find evidence to support a poorer food environment, such as higher concentration of fast food restaurants and a limited selection of fresh produce in grocery stores and markets in low income areas when compared to higher income areas [28-31].

While there is growing evidence to support the deprivation amplification hypothesis in physical activity resources and food outlets, there is also a great deal of variability across studies [32]. This is highlighted by recent systematic reviews of the built environment and obesity, in which the authors conclude that there are few definitive statements to be made when generalizing across studies [9,10,33]. For example, some studies support equal number of resources for physical activity [34] or grocery stores in low-income or black neighborhoods [20] which contrasts other studies that support deprivation amplification [29]. Further, the vast majority of these studies occur in large urban environments, particularly those focused on health disparate populations. In fact, in the review by Feng et al., nearly all the studies, 56 out of 63 included studies, focused on large, urban environments [9]. Therefore, much less is known about potential influences that are particular to small cities and towns or rural areas.

Effectively addressing health disparities requires access to vulnerable and often hard to reach populations in rural as well as urban areas. Community Based Participatory Research (CBPR) may be an effective approach to reach and address health outcomes in these populations [35-37]. Effective CBPR partnerships leverage the collective knowledge, expertise, and resources gained through community-academic partnerships to develop and execute culturally-effective interventions, as prioritized by the community [38,39]. A strong community-academic partnership allows for collaborative conceptualization of the project, a mechanism to disseminate results to the community and a forum for feedback from the community. The inclusion of communities members in built environment assessment research have only recently begun to emerge in the peer-reviewed literature [40], and this study represents one of the first studies to include communities in a participatory research structure.

**Figure 1:**
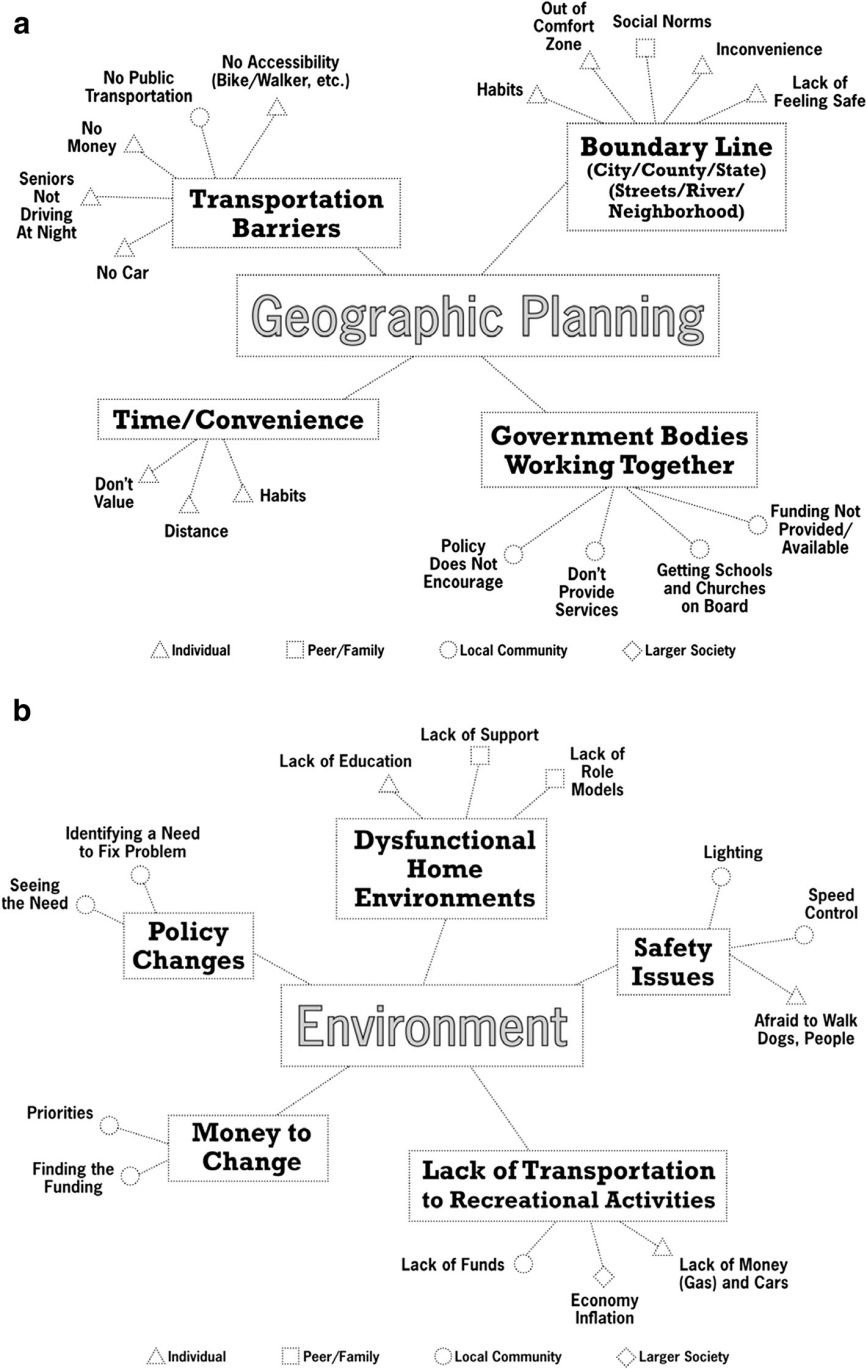
**a. Causal model for geographic influences created during the CPPE workshop. b **. Causal model for environmental influences created during the CPPE workshop.

This built environment study emerged from an ongoing CBPR partnership in the health disparate Dan River Region of south central Virginia and north central North Carolina [41]. This community-academic partnership, formally named the Dan River Partnership for a Healthy Community (DRPHC) initiated in 2009, with a mission to reduce and prevent obesity in the region. In the early stages of this partnership, the community and academic partners hosted a workshop to complete the Comprehensive Participatory Planning and Evaluation (CPPE) process [42,43]. The goal of this workshop was to identify and prioritize obesity and obesity-related causes in the region [41]. The group created 6 causal models for obesity and prioritized immediate action in the form of interventions for 3 causal models (social support for physical activity, nutrition through community gardens and social marketing). A consistent theme across the causal models was the role of environmental and geographical influences on physical activity and nutrition in the region. Factors identified by community stakeholders included access to healthy/unhealthy food, lack of places to be physically active and the ability to use non-vehicle modes of transport (e.g. walk or bus) (See Figure 1a, b) [41].

Using the geographic and environmental causal models as a guideline, with input from the DRPHC members, a plan emerged for a series of geographic and built environment assessment efforts to advance the causal models and to provide data to support various intervention efforts by the DRPHC. Since no comprehensive environmental data was available in the region, the initial step would be to enumerate and geocode the food and physical activity outlets. Following the completion of the enumeration study, phase two would include detailed audits to assess the quality and amenities offered at the outlets. Importantly, under the larger CBPR process, all data collected would be available to the DRPHC members to support other intervention efforts and shared with the public.

This paper is focused on the results of the enumeration study only. The primary aim of this initial phase was to quantify the food and physical activity outlets and to determine if particular areas and/or populations are indeed at a greater disadvantage when considering access to these resources. To guide this inquiry we made several hypotheses which were guided by the current literature and informed by the discussions that occurred during the CPPE workshop and subsequent DRPHC meetings. First, we tested for between block group differences in walkability, food outlets and physical activity outlets. Specifically, we hypothesized that lower income and racially diverse block groups would have fewer food outlets, including fewer grocery stores and fewer physical activity outlets. We further hypothesized that walkability, as defined by a computed walkability index, would be lower in the lower income block groups.

## Methods

Virginia Tech Institutional Review Board has reviewed and approved all study activities.

### Study area

The Dan River region is situated in south central Virginia and north central North Carolina and includes Pittsylvania, Henry, and Caswell counties. Historically, this predominately rural area relied largely on agriculture, manufacturing, and textile mills for its economic foundation. In recent years, much of the manufacturing and textile jobs have disappeared, creating a dire employment situation with unemployment rates between 12–19% which exceeds the state average and national averages [44]. The city of Danville is the largest city in the region, with a population of about 45,000. Danville is the economic hub of the region, where many of the regions’ services are located (e.g. retail, healthcare, higher education centers). An estimated 20% of the residents in Danville are over the age of 65, 44% are black, and 20% are living below the FPL [45,46]. According to the Virginia Health Equity Report, low socio-economic status (SES), rural, and African American populations in the state consistently experience higher mortality rates and poorer health status across a variety of outcomes (e.g. heart disease, cancer, infant mortality, diabetes mellitus) when compared to higher SES, urban and non-black Virginians [4,45]. This is one of the most health disparate regions in the Commonwealth [4,45].

### Data collection

#### Census data

Given that the City of Danville is the only regional city; our initial efforts were restricted to within Danville city limits. The 2000 U.S. census divides Danville in 14 census tracts, and subdivides the tracts into 39 block groups. Socio-demographic and economic characteristics by census tract and block groups were compiled from the 2000 United States Census summary files (SF), in particular, SF1 and SF3 variables. SF1 and SF3 files include variables such as race/ethnicity, the number of female-headed households, education level, and other socio-economic indicators. Census data indicate that the city of Danville is approximately 48% Black and 47% White, therefore race/ethnicities other than black or white were dropped due to empty cells. Given the near even split of race in our study area, we define block group race as predominately Black, predominately White or mixed. After considering the distribution of race within block groups, we conducted sensitivity analyses to determine the appropriate cut-points to classify the block groups. Block groups with a single race > 55% were classified as predominately Black or White. Block groups with a more equal distribution, >45/<55% were categorized as ‘mixed’. Median family income was categorized by deciles. The four lowest deciles for income (1 – 4) were attributed as “low income”, while the four highest deciles (7 – 10) were attributed as “high income”, and the fifth and sixth deciles were attributed as “middle income” [47].

#### Enumeration of food outlets and physical activity resources

To better understand the built environment, a database of food establishments and physical activity resources was created. Retail food outlets and restaurants were identified, enumerated, and classified based on the Nutrition Environment Measures Survey (NEMS) [48,49]. Lists of city and county food permits were matched against lists of outlets from the City of Danville Office of Economic Development. The establishments on the lists were verified using a variety of sources and methods including online searches, telephone calls and drive by visualization to ensure that only those still in operation were included. Further, only establishments that were open to the public were included in this study. Stores were classified into two categories (grocery store and convenience store) and restaurants were classified into three categories (fast casual, fast food and sit down) [48,49]. The City of Danville Office of Economic Development provided initial data on public indoor and outdoor recreation sites. Online business directories provided information on private recreation facilities. Addresses for all food and physical activity outlets were geocoded and mapped.

#### Walkability index

A walkability index was calculated for each block group based on work by Saelens et al. (2003) and Frank et al. (2003) and supported by several empirical studies [12,47,50-54]. This walkability index was well suited for our study as it makes use of publicly available U.S. Census data and road network files, along with other GIS-based data that is typically available from urban planning or transportation departments, even in smaller municipalities [12,47]. Further, there are other studies that support the walkability index and its variants based on locally available data [12,50,52-54].

The walkability index components are defined and calculated as follows. Intersection density is established as the number of true intersections (at least 3 connecting road segments) per block group acre [47]. In addition to removing non-true intersections prior to calculating intersection density, we also removed the non-walkable intersections that result from cloverleaf highway intersections (interconnecting highway on and off ramps). Residential density is defined as the number of residential buildings per residential acre for a block group [47]. Using available land use data, we calculated the acreage of residentially zoned areas per block group as well as the number of building footprints that fell in these areas to determine residential density. Finally, land use mix consists of a diversity score for the square footage of buildings classified under different uses. The classifications available for land use data in our study area resulted in 4 land use categories: institutional, office, residential and retail/commercial [12,47,54]. Land use data are used to classify building footprints into their respective categories. We then calculated the square footage of buildings and summarize them by land use category to generate the land use mix score for each block group using this equation:

(1) $$\text{Land use mix=1–}\left[{\left(\text{Office sq}.\text{ft}./\text{Total sq}.\text{ft}.\right)}^{2}+{\left(\text{Institutional sq}.\text{ft}./\text{Total sq}.\text{ft}.\right)}^{2}+{\left(\text{Residential sq}.\text{ft}./\text{Total sq}.\text{ft}.\right)}^{2}\right]$$

All component values (intersection density, residential density, and land use mix) are converted to z-scores before calculating the walkability index for each block group with the formula from Frank et al [47].

(2) $$\text{Walkability Index=}\left(2\times \text{intersection density}\right)\text{+residential density+land use mix}$$

Following the work of Frank et al. [12,47], we developed a geographic information system (GIS) to calculate a walkability index as a continuous score by census block group for our study area. Data layers used for the walkability index calculation include: census block groups, road network, land use, and building footprints. All data are available through either the US Census Bureau website (http://www.census.gov) or the city of Danville website (http://www.danville-va.gov).

#### Analyses

Statistical analyses were performed using SPSS 18.0™ ArcGIS 9.3™ and GeoDa [55] software. A one-way multivariate analysis of variance (MANOVA) was used to determine if there was a significant difference in the number of food establishments, PA outlets, and walkability by block group of low, middle, and high socioeconomic status. Subsequently, Cohen’s d was calculated to evaluate the effects sizes between block groups.

In addition to testing for differences in absolute numbers, we conducted a spatial analysis to determine patterning among block groups. Spatial autocorrelation describes the distribution of a variable, or the distribution of the relationship between two variables, over space. The pattern of a variable can be clustered, where high (low) values occur near other high (low) values, or dispersed where high (low) values occur near low (high) values, or the pattern could be random [56]. To determine if the spatial patterns of each variable were clustered, we measured the spatial autocorrelation using GeoDa software for walkability, income, fast food outlets per acre (FF/acre), grocery stores per acre (GS/acre), convenience stores per acre (CS/acre), and the percent of block group devoted to parks (% parks). To compare the level of a variable in one block group to that of the second variable in neighboring block groups we conducted a bivariate analysis. This analysis determines if the spatial patterns are clustered, dispersed or random between income and FF/acre, GS/acre, CS/acre, and% parks [57]. For example, the bivariate analysis determines if block groups with high income are next to block groups with a high or low number of fast food outlets per acre. To determine the statistical significance of each Moran’s I calculation, GeoDa calculates a pseudo p-value based on different permutations of a dataset [57]. We ran a series of 999 different permutations with randomized versions of our dataset, and if the Moran’s I for our observed dataset was higher than the randomized Moran’s I calculations 95% of the time, the Moran’s I is considered to be statistically significant (α = 0.05) according to the calculated pseudo p-value [57].

## Results

### Enumeration

In total, there are 49 stores, 160 restaurants and 79 physical activity outlets. Table 1 shows the distribution of the outlets by block group based on income and race categories. The walkability index ranges from −5.64 to 5.23 with a median score of −0.21. Higher index values indicate higher walkability. Block groups with higher scores are more walkable based on this index. The MANOVA (Table 2) shows that only the number of restaurants differed by income for block groups (p = .009). However, Levene’s test for homogeneity of variance was significant (p = 0.00) indicating that equal variance is not assumed between the block groups. Therefore, the post-hoc tests do not show any significance between counts of restaurants by block groups. Other variables did not differ significantly by block group (Walkability Index, p = 0.106; PA outlets, p = 0.572. Count of stores, p = 0.111). However, in some cases the Cohen’s d effect size was large (See Table 2). These large effect sizes suggest that differences may indeed exist between block groups, but there may be a lack of power to detect statistical differences. There were no significant differences based on racial composition of block groups (e.g. predominately white, predominately black, or mixed race; data not shown).

**Table 1:** Enumerated resources by block group

Income*	Race**	**CT-BG**	**Restaurants**				**Stores**		**PA outlets**	Walkability^***^
			**FC**	**FF**	**SD**	**SP**	**GS**	**CS**		
Low	White	CT 12 BG 2	0	0	0	0	0	0	0	−5.26
	Black	CT 3 BG 2	0	1	0	0	1	0	0	4.71
		CT 3 BG 3	0	1	1	0	0	1	5	2.25
		CT 4 BG 1	0	0	0	0	1	2	0	0.89
		CT 4 BG 2	0	0	0	0	1	0	3	−1.78
		CT 5 BG 1	1	1	1	1	2	0	1	4.50
		CT 5 BG 2	0	1	1	0	0	2	3	2.80
		CT 6 BG 1	2	0	0	0	0	0	5	2.64
		CT 6 BG 3	0	0	0	0	0	1	2	2.65
		CT 6 BG 4	0	0	0	0	1	1	1	4.80
		CT 10 BG 1	0	0	2	0	1	1	3	−0.21
		CT 11 BG 1	0	0	0	0	0	0	0	−0.88
		CT 11 BG 2	1	1	0	0	0	0	0	2.95
	Mixed	CT 4 BG 3	0	0	0	0	1	0	1	1.90
		CT 4 BG 4	1	0	1	0	0	0	4	−2.62
Middle	White	CT 2 BG 3	1	2	7	0	1	1	2	1.49
		CT 3 BG 1	0	0	0	0	0	0	2	1.72
		CT 8 BG 1	9	16	26	3	3	3	0	−2.55
		CT 8 BG 2	1	3	3	1	0	2	1	0.00
		CT 9 BG 2	0	0	2	0	0	2	0	−3.18
		CT 10 BG 2	0	0	0	0	0	0	4	−1.20
	Black	CT 2 BG 1	1	10	6	0	0	1	0	−0.52
		CT 6 BG 2	0	0	1	0	0	0	0	3.48
	Mixed	CT 13 BG 1	2	8	5	0	2	5	18	−2.79
High	White	CT 1 BG 2	2	2	0	0	0	1	0	0.60
		CT 1 BG 3	0	0	1	0	0	0	3	3.40
		CT 2 BG 2	0	4	0	0	0	1	2	−0.42
		CT 7 BG 1	0	0	0	0	0	0	5	4.13
		CT 7 BG 2	1	1	1	0	0	0	6	0.33
		CT 7 BG 3	2	3	3	0	0	0	0	5.23
		CT 8 BG 3	1	0	1	0	0	0	0	−3.20
		CT 9 BG 1	0	1	1	0	1	3	1	−3.36
		CT 11 BG 3	0	0	1	0	0	0	2	−4.29
		CT 12 BG 1	0	1	2	0	0	0	0	−5.31
		CT 14 BG 1	0	0	0	0	1	1	0	−5.64
		CT 14 BG 2	0	0	1	0	0	1	3	−4.43
	Mixed	CT 1 BG 1	1	4	2	0	0	2	0	−0.89
		CT 1 BG 4	0	0	0	0	0	0	2	−1.14
		CT 9 BG 3	0	0	0	0	1	1	0	−0.82

CT = census tract; BG = block group.

*Income: Income was categorized into deciles with the four lowest deciles (1–4) categorized as “Low”, the middle two deciles (5–6) categorized as “Middle”, and the highest four deciles (7–10) categorized as High”.

**Race: Race was categorized by the dominant race for the block group. Any block group with a race >55% was classified based on that race. Any block group with a 45–55% race was classified as “Mixed”.

***Walkability Index is based on sum of *z-scores* and ranges from −5.64 to 5.23. Higher positive scores reflect increased walkability.

**Table 2:** Results from MANOVA and effect sizes by block group income

**Variable**	**F**	**Sig**	**Mean**	**SD**	**Cohen’s d**
Walkability Index	2.39	.106			
Low Income			1.4	2.6	
Medium Income			−1.0	2.9	
High Income			−1.4	3.9	
Low to Medium Income					0.87
Low to High Income					0.84
Medium to High Income					0.11
PA outlet (Count)	0.57	.572			
Low Income			1.7	1.8	
Medium Income			2.7	4.4	
High Income			1.3	2.4	
Low to Medium Income					0.19
Low to High Income					0.30
Medium to High Income					0.40
Food Outlet: Stores (Count)	2.34	.111			
Low Income			1.1	0.9	
Medium Income			1.8	2.2	
High Income			0.3	0.8	
Low to Medium Income					0.88
Low to High Income					0.41
Medium to High Income					0.88
Food Outlet: Restaurants (Count)	5.33	.009*			
Low Income			2.2	4.0	
Medium Income			6.2	13.5	
High Income			4.0	2.8	
Low to Medium Income					0.52
Low to High Income					0.39
Medium to High Income					0.23

*While the MANOVA is significant for this variable, the post-hoc tests were not significant based on Levene’s test for equality of variance.

### Spatial analyses

The univariate spatial autocorrelation analysis indicated that the spatial pattern of three variables, walkability, income, and the percentage of convenience stores per acres are statistically significant (Table 3). Since the Moran’s I value is positive in each case, a clustered spatial pattern of each variable is suggested by the analysis, where block groups with high walkability, income, and percentage of convenience stores per acre are clustered near other block groups with high values of those variables.

**Table 3:** **Results of the univariate spatial autocorrelation analysis, *italics *** **= statistically significant (α = 0.05)**

Variable*	**Moran’s I**	**Pseudo p-value**
*Walkability*	*0.50*	*0.001*
*Income*	*0.21*	*0.010-0.017*
Fast Food per acre	0.11	0.067-0.082
Grocery Stores per acre	−0.04	0.459-0.488
*Convenience Store per acre*	*0.19*	*0.014-0.023*
% parks	−0.03	0.519-0.564

*% parks = percent of block group devoted to parks.

According to the bivariate spatial autocorrelation analysis (Table 4), the relationship between income and two variables, percentage of grocery stores per acre and percentage of convenience stores per acre, in neighboring block groups has a statistically significant spatial pattern. Moran’s I in both cases is negative, suggesting that the spatial pattern is dispersed. Block groups with high incomes are next to block groups with a low percentage of grocery stores per acre and a low percentage of convenience stores per acre.

**Table 4:** **Results of the bivariate spatial autocorrelation analysis, comparing each variable with income, *italics *** **= statistically significant (α = 0.05) **

Variable*	**Moran’s I**	**Pseudo p-value**
Fast Food per acre and income	0.08	0.101-0.137
*Grocery Store per acre and income*	*−0.19*	*0.001-0.003*
*Convenience Store per acre and income*	*−0.23*	*0.002-0.007*
% parks and income	0.08	0.080-0.108

*% parks = percent of block group devoted to parks.

## Discussion

While there is growing evidence that living in disadvantaged areas is associated with environments that include reduced access to healthful and affordable food and places to be physically active [8,32,58,59], recent reviews also point out that there is variability across studies which limits the extent to which definitive conclusions may be drawn [9,10,33]. Supporting deprivation amplification in food environments, Morland and colleagues [60,61] found that low income and racially diverse neighborhoods have lower quality and higher priced produce. Other research demonstrates increased density of fast food outlets and higher availability of poor food choices (e.g. fast food) in low income areas [28,62,63]. However, we did not find differences in resource availability for food outlets by block group income. Based on the analyses conducted here, the number of outlets is equally distributed between high and low income block groups. While we found no differences in the absolute number of food outlets between the block groups, there could be variability in the quality and price of food options available to residents.

Similarly, many research studies find support for deprivation amplification related to physical activity resources such that fewer physical activity resources exist in low-income minority communities [8,17,34,59,64]. When considering outlets and opportunities for physical activity, we again found no statistically significant differences in the number available to residents. Estabrooks et al. (2003) found that although the number of facilities was equal, there were fewer low-cost or free resources available in low income areas [34]. Therefore, it may be important to determine if the resources for physical activity in these block groups are equitable in terms of cost and services offered. Additionally, there may be issues related to safety and aesthetics of a space that reduce the likelihood of outdoor activity [13,22,31,59,65-67]. Moore and colleagues [20] found that outside, public areas conducive to physical activity (e.g. parks and trails) are of lower quality, lower perceived safety, broken or inadequate equipment in low income areas [20]. Assessing the quality, condition, safety and general amenities of public parks and trail systems available to residents would be important as well to determine if resources are equitably dispersed. Safety and aesthetics are key determinants for walking behavior and assessing this could provide information for potential strategies to increase walking for fitness, transportation and on short trips.

It is important to note the limitations of this study. This is a small regional city set in a rural health disparate region; therefore, the results found here may not be generalizable to other areas. The fact that we find no differences between block groups could be due to statistical power as there are only 39 block groups in the city. However, this analysis includes all available block groups and all available outlets within the city, not a sample; therefore it represents what is truly available to the residents. Given that fewer studies in the built environment literature are focused in rural environments [9], our methods may be important for other researchers considering rural populations or small to mid-size cities. For the outlets identified in this study, systematic audits to assess features such as quality and prices are currently underway. Further, efforts to enumerate food outlets and physical activity resources in the outlying counties and smaller towns in the region will begin in early 2012, importantly allowing us to explore possible differences between residents of Danville and those in the outlying rural areas. Since our work occurs under a larger CBPR process, this enumeration study represents a critical first phase in building a contextual overview to support on-going efforts to reduce obesity by the DRPHC. To honor the CBPR process, a subcommittee of the DRPHC will be established to assist in determining additional avenues for dissemination of these findings to the region and to determine the overall utility of the data. It is anticipated that the results from this and future geographic and built environment studies in the region will assist the DRPHC in engaging local planning officials to inform policy decisions to positively impact health of all residents in the region.

## Conclusions

As the body of literature on obesogenic environments continues to evolve there is not a ‘typical’ profile that fits all low-income or health disparate populations. Further, conclusions differ across settings, populations and behaviors (e.g. physical activity or food environments). Therefore, efforts to understand and explore the potential influence of the built environment on food availability and choice or physical activity in a given population need to be locally generated. While this smaller lens is not typical for public health, it allows for the creation of a contextual reality regarding food and physical activity environments that is relevant to the population under study. Other community coalitions could consider starting with a similar enumeration approach, as this type of contextual information can inform local stakeholders, policy makers and residents to determine next steps as well as which types of interventions may be most effective in their region. Under the larger CBPR process, this enumeration study advances the causal models set forth by community members to address obesity by providing an overview of the food and physical activity environment in the Dan River Region. This will aid many community-academic partners as they pursue intervention strategies targeting obesity. In light of the CBPR process, the lack of statistical significance of these findings is really a moot point. This data reflects the context of available resources from which to plan and implement future health promotion activities in the region for this group of community stakeholders.

## Competing interests

The authors declare that they have no competing interests.

## Authors’ contributions

JH, KK and JZ conceptualized and drafted the paper. Each author contributed to further development and revisions of the paper, and approved the final submission. Each author assumed a unique role in execution of this research including: JH and JZ secured grant funding for the project and conceptualized the study design; CL and CC each participated in data acquisition and analysis; KK and CL provide GIS and spatial expertise; CC provided project and data management support. All authors read and approved the final manuscript.
